# Identification of shedders of elephant endotheliotropic herpesviruses among Asian elephants (*Elephas maximus*) in Switzerland

**DOI:** 10.1371/journal.pone.0176891

**Published:** 2017-05-03

**Authors:** Mathias Ackermann, Jean-Michel Hatt, Nelli Schetle, Hanspeter Steinmetz

**Affiliations:** 1 University of Zurich, Vetsuisse Faculty, Institute of Virology, Zurich, Switzerland; 2 University of Zurich, Vetsuisse Faculty, Clinic for Zoo Animals, Exotic Pets and Wildlife, Zurich, Switzerland; 3 Walter Zoo, Gossau, Switzerland; University of Melbourne, AUSTRALIA

## Abstract

Elephants, particularly Asian (*Elephas maximus*), are threatened by lethal elephant hemorrhagic disease (EHD) due to elephant endotheliotropic herpesviruses (EEHV). At least five of seven known EEHV types have been associated to EHD, with types 1, 4, and 5 predominantly affecting Asian elephants. In Switzerland, at least three Asian elephants have been lost due to EHD but nothing is known about the present EEHV1 circulation. Moreover, the prevalence of other EEHV types has never been assessed. Intermittent shedding of EEHV can be monitored through collecting trunk secretions and analyzing them by PCR methods that discriminate the different EEHV types. To identify EEHV shedders, seven of eight Asian elephants in a Swiss zoo were trained to provide trunk wash samples. These were collected at intervals over a period of four months and tested by PCR for presence of EEHV1 through 6. Moreover, the quality of each sample was assessed by testing for the elephant TNF-alpha gene. Overall, 57% of the samples were valid with five of seven participating elephants identified as EEHV shedders. Two of those shed virus only once, whereas the other three, all closely related among each other, shed virus on multiple occasions. One of the frequent shedders had been in very close contact to all of the three EHD victims. Therefore, we speculate that this particular animal may represent the virus source in all three cases. However, when subtyping was conducted, the presently circulating virus was identified as EEHV1B, while the virus subtype causing EHD had been 1A in all three cases. In addition to four animals excreting EEHV1, a recently introduced animal was observed to shed EEHV3/4. We suggest that the policy of trunk washing to identify and characterize EEHV-shedders is to be endorsed in zoos with ongoing or planned elephant breeding programs.

## Introduction

Asian elephants (*Elephas maximus*) represent an iconic but endangered animal species that faces the challenge of lethal elephant hemorrhagic disease (EHD) associated with infection with elephant endotheliotropic herpesviruses (EEHV)[1–6]. In Switzerland, at least three individual elephants have been lost to EHD. The first case in 1988, represents the first one world-wide that has been linked to an acute herpesvirus infection [7]. The other two cases, 1999 and 2003, have been confirmed by histopathology and demonstration of EEHV1 DNA in various tissues (unpublished data). Death due to EHD typically occurs in young elephants and the disease is known to occur acutely and progress rapidly, leading to death after one to seven days [7, 8].

Unfortunately, it has hitherto not been possible to propagate any type or strain of EEHV in cell culture. Therefore, knowledge about these viruses, their pathogenesis, and epidemiology lags far behind the understanding of other herpesviruses. However, in recent years, genomic sequence analyses have led to the identification of at least 6 different types of these viruses, which could be associated to EHD. These viruses were designated EEHV1 through 6 [8–11]. These six types may be further divided into three pairs, with one member of each pair affecting either Asian or African elephants (*Loxodonta africana*)[2, 5]. Accordingly, EEHV1, 4, 5 affect Asian elephants, whereas their counterparts EEHV6, 3, 2 represent viruses of African elephants. Moreover, these viruses may be divided into an AT-rich branch (EEHV1, EEHV2, EEHV5, EEHV6) and a GC-rich branch (EEHV3, EEHV4). They have all been assigned to the taxonomic genus *Proboscivirus*, so far the sole member of a newly proposed subfamily *Deltaherpesvirinae* [4, 5]. Based on this newly acquired knowledge of sequences, new diagnostic PCR-assays were developed, which allowed not only detection of EEHV but also immediate type discrimination [12–14]. Thanks to such assays, it was also noted that various EEHV types were shed intermittently through trunk secretions [13]. Although these genome-targeting PCR assays cannot discriminate between EEHV in a latent or actively replicating state, the intermittent detection of these viruses in trunk washes has been linked to episodes of viral excretion. Consequently, detection of EEHV DNA in a trunk wash sample is indicative for an animal that presently excretes EEHV, whereas lack of detection may indicate that the corresponding animal is either not infected or may harbor a particular type of EEHV in a latent, rather than a replicative state.

Due to the confirmed EEHV1 cases diagnosed in the past, we assumed that at least EEHV1 circulated among our Asian elephant population in Switzerland. However, data about EEHV–carriers and–shedders among those animals were hitherto not available but might be very important to know for adapting future disease prevention measures. Moreover, the presence or absence of the other five types of EEHV has also not been determined.

To address these issues, a special trunk wash collection program was initiated in one of the two captive Asian elephant herds in Switzerland. The aim was to monitor EEHV excretion at weekly intervals over a period of four months.

## Materials and methods

### Ethic statement and animals

This study was carried out in strict accordance with the Swiss regulations for animal experimentation. The project was approved by the Research Committee of Zurich Zoo. No further licenses were necessary as the sampling of trunk washes is part of the routine prophylactic health monitoring program at Zurich Zoo.

The study did not require anesthesia, euthanasia, or any kind of animal sacrifice.

The animals investigated in this study belonged to a single herd of Asian elephants kept in a Swiss zoo (Table 1). Seven of eight animals were included in the study.

**Table 1:** Participating animals.

Identification	Gender	Born	Comment
Z2A	Male	1969 in Thailand	1971 transfer to UK zoo; 1981 transfer to Swiss zoo; father to all three Swiss EHD cases
Z2B	Male	2004 in Germany	2014 transfer to Swiss zoo
Z2C	Female	1975 in Sri Lanka	1976 transfer to Swiss zoo; mother to case 2, case 3, and to Z2D
Z2D	Female	2005 in Swiss zoo	Young of Z2C and Z2A
Z2E	Female	1986 in Burma	1988 transfer to Swiss circus; 1999 transfer to Swiss zoo; mother to Z2F and Z2G
Z2F	Female	2002 in Swiss zoo	Young of Z2E and Z2A
Z2G	Female	2014 in Swiss zoo	Young of Z2E and Z2A

### Trunk wash samples

Individual trunk wash samples were obtained as part of the preventive health program according to the RECOMMENDATIONS FOR THE DIAGNOSIS, TREATMENT AND MANAGEMENT OF TUBERCULOSIS (Mycobacteria tuberculosis) IN ELEPHANTS IN HUMAN CARE 2015 (https://assets.documentcloud.org/documents/1694827/elephant-tb-guidelines-2015.pdf, accessed 23.02.2015). Briefly, the elephant held its trunk with its tip upwards. Then, the handler instilled approximately 100–200 mL sterile saline into both nostrils. The animal then lifted its trunk tip as high as possible to help the fluid flow as far into the trunk as possible. After 15–30 sec, the elephant lowered its trunk tip to allow the fluid to drain into a bucket. Additionally, the elephant was asked to exhale into the bag during this collection phase of the procedure. The sample was then transferred into two sterile plastic jars. Samples were submitted for analysis within 4 to 8 hours.

### Formaldehyde-fixed samples

Thin sections from formaldehyde-fixed myocardium (containing inclusion bodies) of all three previous Swiss cases of EHD, were kindly provided by Dr. Franco Guscetti, Institute of Veterinary Pathology, VSF, University of Zurich, Switzerland. The identification numbers were: S88-2399.5, S99-2095.4, S03-2084.6.

### DNA extraction

Upon receipt, trunk washes were centrifuged at 1500 X g for 10 min at room temperature. The supernatant was discarded and the pellets were either processed immediately or stored at −20°C prior to DNA extraction. The Qiagen (Hombrechtikon, Switzerland) DNeasy Blood & Tissue Kit was used for DNA extraction, essentially according to the protocols of the supplier. DNA was eluted in a volume of 60 μL and analyzed using the Nanodrop spectrophotometer. All DNA samples were stored at −20°C until further analysis.

### Real-time PCR

Each sample was subjected to 6 individual Rt-PCR assays, namely for EEHV1, EEHV2, EEHV3/4, EEHV5, EEHV6, and TNF. All real-time PCRs were done essentially as previously described [12–14]. Primers (Microsynth, Balgach, Switzerland) and probes (Lifetechnologies, Switzerland) as well as target genes are listed in Table 2. The Asian elephant tumor necrosis factor (TNF) α gene was used as an indicator for the presence of elephant cells in the sample and as an internal amplification control for DNA under the current PCR conditions [12]. A sample was considered as "valid" only if a Ct value prior to cycle 40 was achieved with the TNF-PCR.

**Table 2:** Primers and probes used in this study.

Target	Oligo^a^	5' to 3' sequences and labels^b^
EEHV1	P-MDBP^c^	6FAM-CAG CAC ACC GCA AAA CCA AAA AAT CTT AAA-MGB/NFQ
	F-MDBP	CGA TGA TAC CCG ATC CCT AGT C
	R-MDBP	GGC GCC GAA GCT TAG ATG
EEHV2	P2-POL^d^	6FAM-CGA CCA CGA AAG AAT A-MGB/NFQ
	F2-POL	CCC AGG GAC GCC AGT TAC TT
	R2-POL	CCA ATC GTT AAA TCT CTC GCA
EEHV3/4^e^	P-TER^f^	6FAM-CAC GTG ATC GCG TCC-MGB/NFQ
	F-TER	TGG GCT TAT GTA ATC GGT AGC
	R-TER	CGT GTG CGA GGA GCA CTT ATA T
EEHV5	P5-POL^g^	6FAM-AGC CGT GAG AGA AA-MGB/NFQ
	F5-POL	CCT GGT TGG CGG AAA GAA
	R5-POL	GCA TCA AAG GGT CAC TAC ACT GTT
EEHV6	P6-POL^h^	6FAM-CGT GTT TAC CGA TAG CC-MGB/NFQ
	F6-POL	AGG CGT CTC AAA GGG TAT GTT
	R6-POL	TCC CTG AGC GGT GAC AGA TT
EEH1A	F-E36^i^	TCC AGG GAT TTC TCC AGT TG
	R-E36	GCC ACC TTC TTC TGC TTT TG
Elephant	P-TNF^j^	VIC-CCA GCT AGA GAA GGG T-MGB/NFQ
	F-TNF	CCC ATC TAC CTG GGA GGA GTC T
	R-TNF	TCG AGA TAG TCA GGC AGA TTG ATC

^a^ P = probe; F = forward primer; R = reverse primer

^b^ FAM and VIC were used as reporter dyes; Minor Groove Binder and Non-Fluorescent Quencher (MGB/NFQ) were added to stabilize the probe for a more robust assay

^c^ Major DNA-binding protein gene (U41) of EEHV1

^d^ DNA polymerase gene (U38) of EEHV2

^e^ The target sequences for EEHV3 and EEHV4 are identical

^f^ Terminase subunit 1 (U60) of EEHV3 and EEHV4

^g^ DNA polymerase gene (U38) of EEHV5

^h^ DNA polymerase gene (U38) of EEHV6

^I^ primer-binding sequences conserved among EEHV1A and B, bracketing a sequence that contains numerous differences between the two (see text).

^j^ TNF-alpha gene of the Asian elephant

Each individual PCR was run in a volume of 20 μL, which included 5 μL of DNA, 1 μL of 20x assay mix (to give a final concentration of 0.9 μM for each primer and probe), 4 μL Taqman Master Mix and 10 μL water. After 2 min at 50°C and an initial denaturation at 95°C for 2 min, the samples were cycled 40 times for 15 sec at 95°C followed by 1 min at 60°C. The threshold was set to a maximum of 0.08. A sample which surpassed this threshold prior to cycle 40 was considered positive, otherwise it was considered negative.

For fear of contamination, we did not carry positive controls along with each test for detecting a particular virus. While this may cause scrutiny with regard to the non-detection of certain virus types, it strengthens also the value of virus detection. Notably, we never re-opened any sample after real-time PCR in order to strictly avoid any contamination of the laboratory with amplified DNA. In our opinion, the validation of the samples by amplification of the elephant TNF gene to confirm the presence of suitable and amplifiable DNA more than counter balances the lack of positive controls for each virus.

### Conventional PCR

A novel PCR was designed to discriminate between EEHV1A and EEHV1B. The target, the E36 gene ([10]; also designated U79 by [11]), was selected for its vicinity to E36A (also termed EE6 [11]). E36A/EE6 has been predicted to be an intact gene in EEHV1A but truncated in EEHV1B [11]. Upon alignment of the known EEHV1A and EEHV1B sequences in this region, a relatively short 120 bp sequence within E36/U79 was identified that contained signature differences between EEHV1A and 1B. The primers used are listed in Table 2. The PCR conditions were as follows:

The PCRs were run in a volume of 20 μL, comprising 5 μL template, 10 nm dNTP, 5x Phusion GC buffer and 2 U of Phusion High-Fidelity DNA Polymerase (Thermo Scientific) and each primer at a concentration of 0.5 μM. After an initial denaturation for 2 min at 98°C, the samples were cycled 39 times at 98°C for 10 sec, 58.4°C for 20 sec, and 72°C for 7 sec. The run was completed with 10 min at 72°C before cooling to 10°C. The PCR product was evaluated for its size (expected approximately 120 bp) by agarose gel electrophoresis before being extracted from the gel for cycle sequencing by a commercial company (Microsynth, Balgach, Switzerland).

## Results

### Animals

During the sampling, all the animals remained clinically healthy. Importantly, none of the animals showed any clinical signs of EHD at any time throughout our study.

### Sampling

Overall, samples from 7 individual elephants were collected (Table 3). Since sampling depends largely on the training status of the individual, the sampling success varied considerably but improved over time. Over a period of four months, all animals were sampled 14 times, amounting to between 6 and 11 valid samples per participating animal.

**Table 3:** Sampling, sample validity, EEHV detection.

Sampling	Z2A	Z2B	Z2C	Z2D	Z2E	Z2F	Z2G
**Sep 22**	iv	iv	iv	iv	iv	iv	iv
**Sep 29**	iv	iv	iv	iv	iv	iv	iv
**Oct 7**	v/EEHV1	v/neg	v/neg	v/neg	v/EEHV1	v/EEHV1	v/EEHV1
**Oct 15**	iv	v/neg	iv	iv	v/EEHV1	iv	iv
**Oct 20**	iv	iv	iv	v/neg	v/EEHV1	v/EEHV1	v/EEHV1
**Nov 2/4**	iv	iv	v/neg	v/neg	v/neg	v/neg	v/neg
**Nov 12**	v/neg	iv	iv	iv	v/neg	v/neg	v/neg
**Nov 17**	v/neg	v/neg	iv	iv	v/neg	v/neg	v/neg
**Nov 23**	iv	v/neg	v/neg	v/neg	v/EEHV1	v/neg	v/EEHV1
**Dec 2**	v/neg	v/neg	v/neg	v/neg	v/neg	v/neg	iv
**Dec 8**	iv	v/neg	v/neg	iv	v/neg	iv	iv
**Dec 15**	v/neg	v/neg	v/neg	v/neg	v/neg	v/neg	iv
**Jan 4**	iv	v/neg	v/neg	v/neg	iv	v/EEHV1	iv
**Jan 20**	v/neg	v/EEHV3/4	iv	iv	v/neg	iv	iv
**Valid**	6	10	7	7	11	9	6

iv = invalid sample, TNF not amplified

v = valid sample, TNF amplified

EEHV number = Type of EEHV detected

neg = EEHV not detected

Valid = total of valid samples per animal

### Sample validity

Trunk washes may contain a lot of contaminating materials, such as food particles and bacteria, all of which may provide DNA upon DNA extraction. Moreover, herpesviruses are known to be highly cell associated. Therefore, we considered the presence of elephant cells in the trunk washes as an important indicator for a valid sample. To demonstrate that elephant cells had actually been in the sample, we took advantage of a previously established method to amplify sequences from the elephant TNF alpha gene by quantitative PCR [12]. Samples that provided a signal with this PCR were considered as valid in terms of containing elephant cells as well as for being extracted to yield an amplifiable DNA template. Based on these conditions, we obtained 57% valid samples (Table 3).

### Detection of EEHV shedding

As documented in Table 3 five of the seven animals were identified as EEHV shedders. Four elephants shed EEHV1, whereas one additional elephant shed EEHV3/4. Just one shedding period was observed for each of the two male elephants. Three of the five female elephants experienced shedding episodes more frequently, i.e. three to four episodes were observed with each one of them throughout the observation period.

### Determination of the EEHV1 subtype

Two genetically distinct subtypes of EEHV1 have been defined, subtype 1A and subtype 1B, respectively [3, 15]. One of the major differences between the two has been mapped to a gene named EE6, which codes for a distinct protein in EEHV1A, whereas it is disrupted in EEHV1B [11]. However, a neighboring gene, U79, is also quite conserved between the two subtypes. We selected two highly conserved sequences within U79 as primer-binding regions in order to produce an approximately 120 bp PCR product, whose sequence would either match to EEHV1A or EEHV1B. According to the alignment, there are 12 differences that discriminate between subtype 1A and subtype 1B within the sequence between the two primers. Subtype 1A has 86 nucleotides in this sequence, three of which have no counterpart in Subtype 1B with 83 nucleotides. Of the remaining nucleotides, 9 differ between the two subtypes.

**Fig 1:**
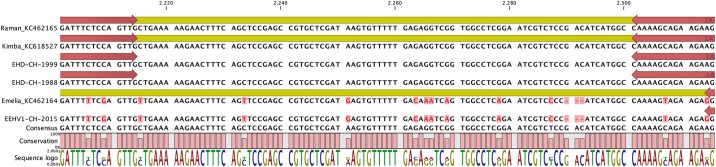
Determination of the EEHV1 subtype. Alignment of the genomic sequences of EEHV1A and EEHV1B with the PCR products from two historic Swiss cases of EHD (EHD-CH-1988 and EHD-CH-1999) as well as with a recent Swiss EEHV1 isolate (EEHV1-CH-2015). Kimba and Raman represent two different prototype viruses of EEHV1A, whereas Emelia is the prototype virus strain for EEHV1B. Conserved sequences are lettered in black; non-conserved sequences are lettered in red. The map positions of the primer sequences are indicated by arrows. (Figure exported from CLC Main Workbench 7).

Indeed, one of the trunk washes from animal Z2G gave a very strong signal for both TNF and EEHV1 DNA and provided a strong band with the newly developed EEHV1A/B-PCR (S1 Fig). This band was extracted and subjected to cycle sequencing. Moreover, formaldehyde-fixed tissue from the previous three Swiss cases of EHD were made available to us to extract DNA for the same PCR assay. All three samples gave a band of the expected size, which was then excised and subjected to cycle sequencing. The 1988 and 1999 cases provided overlapping sequence information from both ends of the respective PCR products (S2 and S3 Figs), whereas the 2003 case could be sequenced only from one side, providing an incomplete result (therefore not included in Fig 1; data presented in S4 Fig). However, as far as available, the sequences of the three Swiss EHD cases were identical among each other but different from the sample obtained from animal Z2G during the present study (designated as EEHV1-CH-2015).

The sequences were then aligned to be presented in Fig 1, which includes also an alignment between the two prototypes of subtype 1A (Kimba, GenBank: KC618527.1; Raman, GenBank: KC462165.1) and the prototype of subtype 1B (Emelia, GenBank: KC462164.1).

In the case of the three Swiss EHD cases, each single one of the 12 predicted ambiguities between the subtypes was identical to the sequences published for the two prototypes of EEHV1A (Raman, Kimba). In contrast, EEHV1-CH-2015, detected in a three-year-old calf, matched exactly to the sequence of subtype 1B (Emelia).

These results suggest that at least two different types of EEHV presently circulate among these elephants: EEHV1, which was expected due to the previous cases of EHD and EEHV3/4, which is new to the zoo. Subtyping of the presently circulating viruses was possible only in one instance and the subtype determined was EEHV1B. In contrast, the myocardium samples from the three EHD cases all comprised EEHV1A DNA.

## Discussion

Throughout the past 30 years, at least three Asian elephant calves have been lost to EHD in Switzerland. Many more cases have been observed worldwide, both in zoo-kept and also in wild-living elephants [1]. Due to the general failure to propagate the associated viruses in cell culture, progress on the knowledge about EEHV1 epidemiology and the pathogenesis of EHD has been slow. However, one important breakthrough has been achieved through the detection of EEHV-shedding in trunk washes [13]. A second important development was the ability to determine genomic sequences of the various EEHV-types, which resulted in the distinction of at least 6 EHD-associated types of EEHV [2, 8–11, 14]. Moreover, a 7^th^ type of EEHV has now been identified but so far has not been associated with EHD [1]. Based on this sequence information, specific real-time PCRs for the detection of the different EEHV-types has been developed [14]. Of particular value was the PCR for the detection of the elephant TNF alpha gene, which served as a positive control for the presence of elephant cells in the trunk wash and for DNA templates suitable for PCR amplification [12, 13].

The Asian elephants in this study underwent training to provide suitable trunk washes, which were analyzed for the presence or absence of EEHV-DNA.

The salient features of these studies are as follows:

1. Among 7 Asian elephants tested over a period of four months, 5 individuals were identified as shedders of EEHV. Although no recent cases of EEHV-hemorrhagic disease have been reported, our data provides evidence that EEHV still circulates among the Asian elephants in Switzerland.
2. After two instances of invalid sampling, it was interesting to observe that the frequency of providing valid samples increased to a final value of 57%. The increase of valid sampling was attributed to a policy, which preferentially selected the murky washing contents for laboratory analysis. Of course, a number of invalid samples still remained, which was tolerable in the present screening context. In these instances, it remains unknown, whether the individual shed virus at the particular time or not. Thus, the sampling methods described here may not be suitable for studies that might require more frequent and abundant detection of virus or viral DNA, which may be important to measure the success of antiviral treatment [16].
3. In four of the shedders, DNA of EEHV1 was detected, whereas one individual shed EEHV3/4. The three previous cases of documented EHD in Switzerland have been associated to EEHV1 ([7] and unpublished data). This study represents, however, the first time to record detection of EEHV3/4 among Swiss elephants. Although the PCR used does not discriminate between EEHV3 and EEHV4, we assume that EEHV4 had actually been detected, since EEHV3 is associated to African elephants, whereas EEHV4 is considered as a virus of Asian elephants [2]. Yet, further investigation into identifying the current type is currently under way.
4. Our assays had been designed to detect all 6 known types of EEHV. Therefore, it is important to state that we did not detect any shedders of EEHV2, EEHV5, or EEHV6 throughout our study. Two major reasons may account for this observation: (i) shedding of any of these viruses did not occur throughout the period of observation, and (ii) EEHV6 and EEHV2 are considered viruses of African, rather than Asian elephants [2, 5].
5. No evidence of shedding was found in one pair of elephants, a mother (Z2C) and her daughter (Z2D), suggesting that they may be regarded as rare-shedders or non-shedders, on condition that they are actually infected at all. Two bulls, one (Z2A) contributing 6 valid samples, the other (Z2B) 10 valid samples, were identified as one-time-shedders, which is certainly not frequent but gives proof that they carry a virus that can be excreted. It would be interesting to know, whether or not their hormonal status may influence the frequency of shedding. It may be worthwhile to note that none of the bulls has been in musth throughout the period of sampling.
6. Three elephant cows, a mother (Z2E) and two of her daughters (Z2F, Z2G), shed virus intermittently for three to four times within the period of observation. We speculate that frequent shedding may be an inheritable trait and may thus have a genetic link. It is well known that herpesviruses may behave differently in genetically different individuals [17–19]. In the case of the Chelonid herpesvirus 5, which causes fibropapilloma in marine turtles, it has even been suggested that "super-shedders" may play a major role in the transmission and maintenance of the agent in nature [20]. It will be interesting to determine in the future whether or not this is also the case with EEHV in elephants.
7. It is important to note that two of the three cases of EHD (cases 2 and 3) in Switzerland occurred in this particular zoo, whereas the very first case (1988) occurred in a touring circus [7]. The only four-time-shedder, Z2E, mother of the two other frequent shedders, had been a freshly imported novice on tour with the same circus, when case 1 had occurred. Moreover, she had been introduced to the zoo’s elephant herd in 1999, approximately one month before a calf of the non-shedder Z2C succumbed to EHD (case 2). Moreover, she had also been in continuous close contact with a male offspring of Z2C, before that calf also succumbed to EHD in 2003 (case 3). Nevertheless, it is of high interest to further investigate this issue by systematically discriminating the subtypes 1A and 1B in future trunk wash samples from Z2E. Together, these data lead us to hypothesize that offspring of non-shedders are particularly in danger of succumbing to EHD, provided that frequent shedders are present in the same herd.
8. The youngest member of the elephant herd (Z2G) provided the sample with the highest load of EEHV DNA among all the samples taken within the observation period (details not shown). Using this sample for a further PCR that had been developed to distinguish between the two subtypes of EEHV1, namely subtype 1A and subtype 1B, it turned out that the amplified sequences exactly matched with the sequences from the subtype 1B prototype strain Emelia [11]. Unfortunately, there was no material left to also subtype the other EEHV1-positive trunk wash samples. However, when historical samples from the three Swiss EHD cases were subjected to the same PCR, all three products aligned perfectly with the 1A subtype strains Raman and Kimba [10, 11]. Unfortunately, these historical samples did not provide longer PCR products than the ones described here. Particularly, the thymidine kinase and vOX2 loci would have been of great interest [1]. This failure may be attributed to long-time storage as formaldehyde-fixed tissues. Although circumstantial evidence suggests that Z2E was probably the virus source for all three Swiss EHD cases, we cannot yet confirm that she is actually a carrier and shedder of the 1A subtype. It will be very important to address this question in the future.
9. The detection of EEHV3/4 DNA in the trunk wash from Z2B, the younger of the two bulls, came as a surprise. The amount of DNA recovered from the positive sample was insufficient to further investigate into the actual virus type. With the present assay, EEHV3 cannot be distinguished from EEHV4. However, since EEHV4, rather than EEHV3, is known to circulate among Asian elephants, we speculate that the present virus may represent EEHV4 [2, 5]. Notably, EEHV3 and EEHV4 represent viruses that are markedly divergent from EEHV1, although they have also been detected in the context of elephant hemorrhagic disease [1, 8]. They belong to the GC-rich branch of the Probosciviruses and are believed to have a less selective organ tropism than their AT-rich counterparts [8]. Apart from elephant hemorrhagic disease, ascites, widespread visceral edema, petechiae, and retinal vascular damage have been associated with EEHV3 and EEHV4 infections [8]. The bull, in which EEHV3/4 shedding has been detected, was only recently introduced to the zoo. Knowing that he may transmit a new virus to his novel mates requires special attention.

## Conclusions

With the present study, we provide data for the first time about the excretion of EEHV1 and EEHV3/4 among Asian elephants in Switzerland. Four of seven animals in the study were identified as shedders of EEHV1. With three to four shedding episodes within a time period of just three months, three individuals, all closely related to each other, were classified as frequent shedders. Presently, we found evidence for a circulating subtype 1B, while all three Swiss cases of EHD could be attributed to the subtype 1A. One animal, a recently introduced bull, was identified as a shedder of EEHV3/4, which seems to be a novel virus among Swiss elephants.

The present data allow also a retrospective evaluation of the Swiss EHD cases.

Shedding of EEHV1 was not detected throughout our study, in the mother (Z2C) of two of the three Swiss EHD victims. Consequently, she can be classified as rare-shedder or non-shedder, a condition that may well lead to low antibody production and, subsequently, to low transfer of maternal antibodies to her offspring, which, in turn, might explain the susceptibility of her offspring to EHD.

In contrast, Z2E was identified as a frequent shedder of EEHV1 and had been in close contact to all three EHD victims. Therefore, the most likely scenario is that Z2E, born in Burma (today known as Republic of the Union of Myanmar), carried EEHV1 with her, when she was imported to Switzerland, where she met the later case 1 animal. Subsequently, Z2E most likely also infected cases 2 and 3, which led to their deaths. The introduction and maintenance of a frequent EEHV1-shedder, various forms of stress as well as lack of knowledge about EEHV and its pathogenesis and epidemiology were most likely the reasons for the three cases of EHD among Swiss elephants. The presently established diagnostic tools in combination with appropriate management and therapeutic considerations will hopefully make it possible to avert such cases in the future.

## Supporting information

S1 Fig Assembly of sequenced PCR product from animal Z2G. The PCR product was obtained by using 1A primers and EEHV1-positive trunkwash from animal Z2G as template. Cycle sequencing was done using the same primers. The obtained forward and reverse sequences were aligned against the EEHV1B reference (Emelia). **Mind**: the 1A primers each comprise two internal mismatches against the 1B sequence.
(TIF)

S2 Fig Assembly of sequenced PCR product from EHD case 1, 1988. The PCR product was obtained by using 1A primers and DNA from EEHV1-positive, formaldehyde-fixed myocardium (S88-2399.5) as template. Cycle sequencing was done using the same primers. The obtained forward and reverse sequences were aligned against the EEHV1A reference (Kimba).
(TIF)

S3 Fig Assembly of sequenced PCR product from EHD case 2, 1999. The PCR product was obtained by using 1A primers and DNA from EEHV1-positive, formaldehyde-fixed myocardium (S99-2095.4) as template. Cycle sequencing was done using the same primers. The obtained forward and reverse sequences were aligned against the EEHV1A reference (Kimba).
(TIF)

S4 Fig Assembly of sequenced PCR product from EHD case 3, 2003. The PCR product was obtained by using 1A primers and DNA from EEHV1-positive, formaldehyde-fixed myocardium (S03-2084.6) as template. Cycle sequencing was done using the same primers. The obtained reverse sequence was aligned against the EEHV1A reference (Kimba). **Mind**: a forward sequence was not obtained.
(TIF)
