# Supplementary figures and images for: Identification of shedders of elephant endotheliotropic herpesviruses among Asian elephants (Elephas maximus) in Switzerland
Source: PLoS One. 2017 May 3;12(5):e0176891. doi: 10.1371/journal.pone.0176891 (PMC5415103; doi:10.1371/journal.pone.0176891)

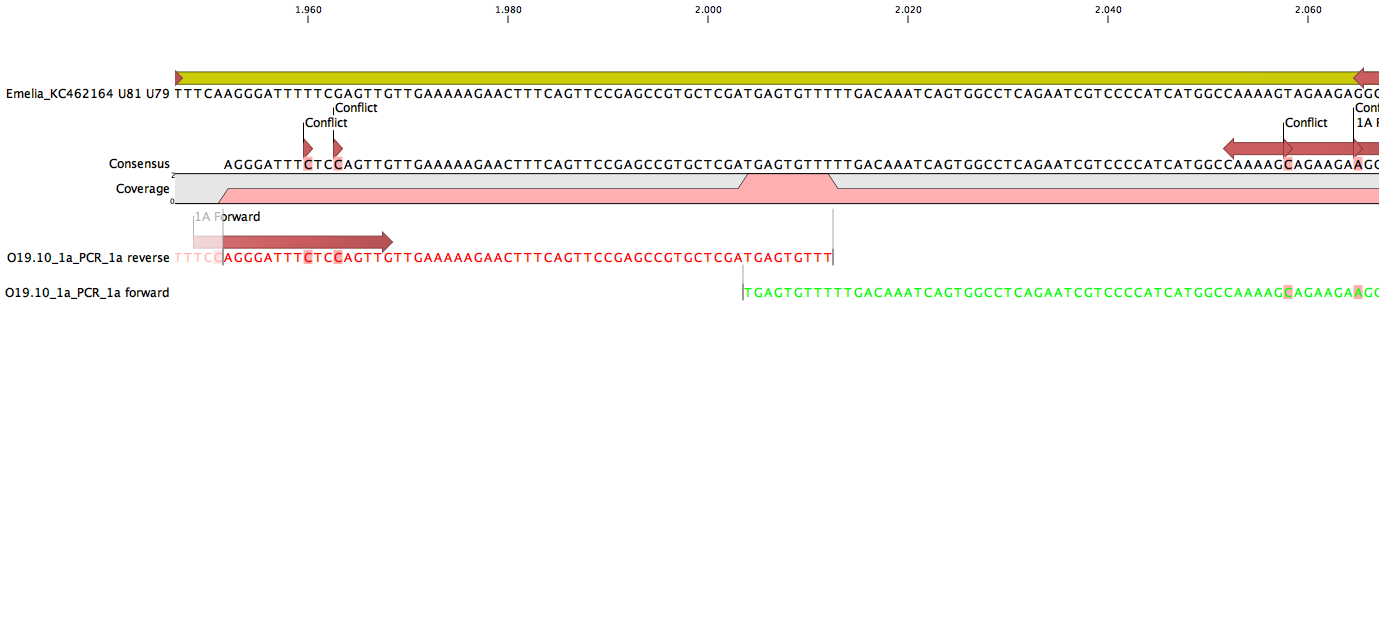

Supplement: S1 Fig — The PCR product was obtained by using 1A primers and EEHV1-positive trunkwash from animal Z2G as template. Cycle sequencing was done using the same primers. The obtained forward and reverse sequences were aligned against the EEHV1B reference (Emelia). Mind: the 1A primers each comprise two internal mismatches against the 1B sequence. (TIF) [file pone.0176891.s001.tif]

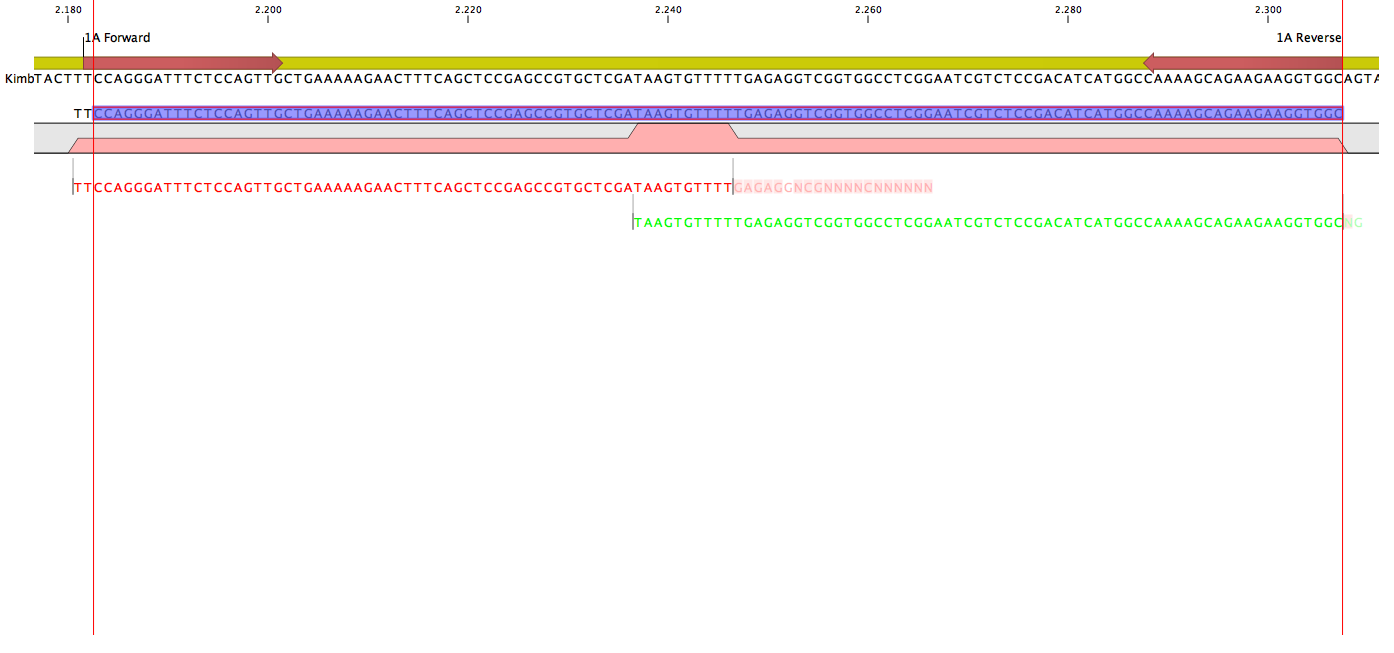

Supplement: S2 Fig — The PCR product was obtained by using 1A primers and DNA from EEHV1-positive, formaldehyde-fixed myocardium (S88-2399.5) as template. Cycle sequencing was done using the same primers. The obtained forward and reverse sequences were aligned against the EEHV1A reference (Kimba). (TIF) [file pone.0176891.s002.tif]

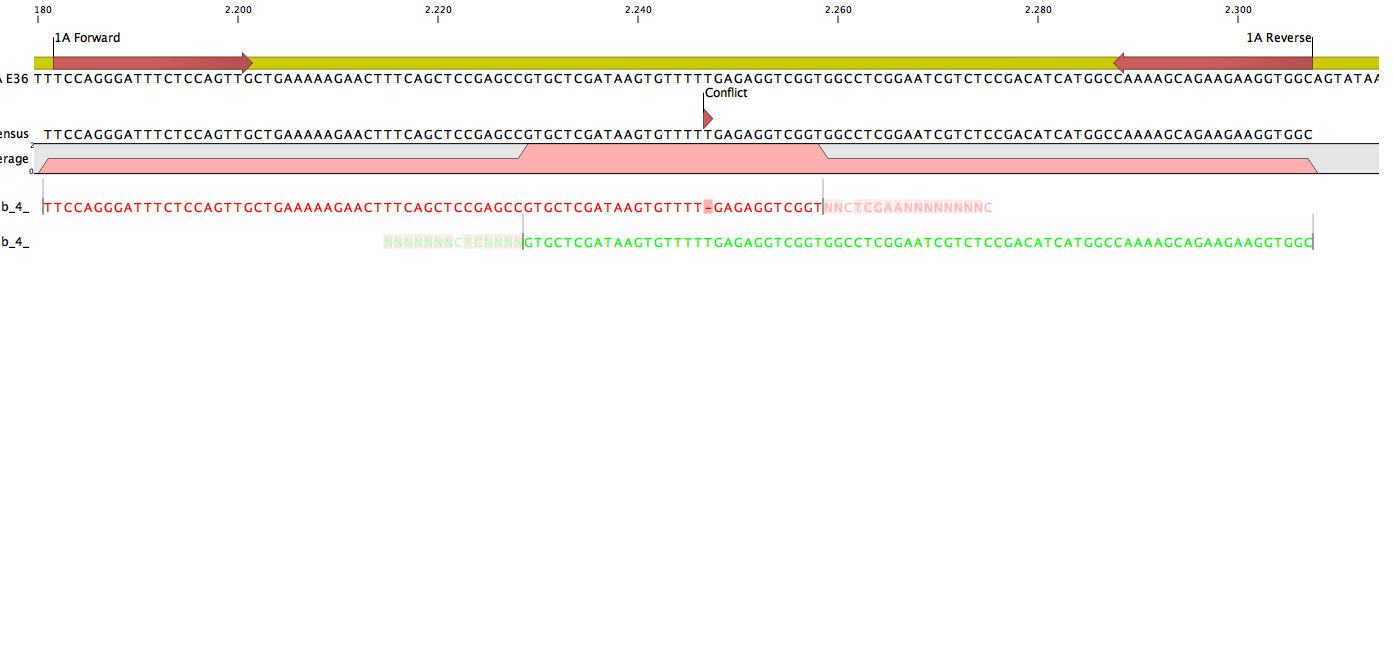

Supplement: S3 Fig — The PCR product was obtained by using 1A primers and DNA from EEHV1-positive, formaldehyde-fixed myocardium (S99-2095.4) as template. Cycle sequencing was done using the same primers. The obtained forward and reverse sequences were aligned against the EEHV1A reference (Kimba). (TIF) [file pone.0176891.s003.tif]

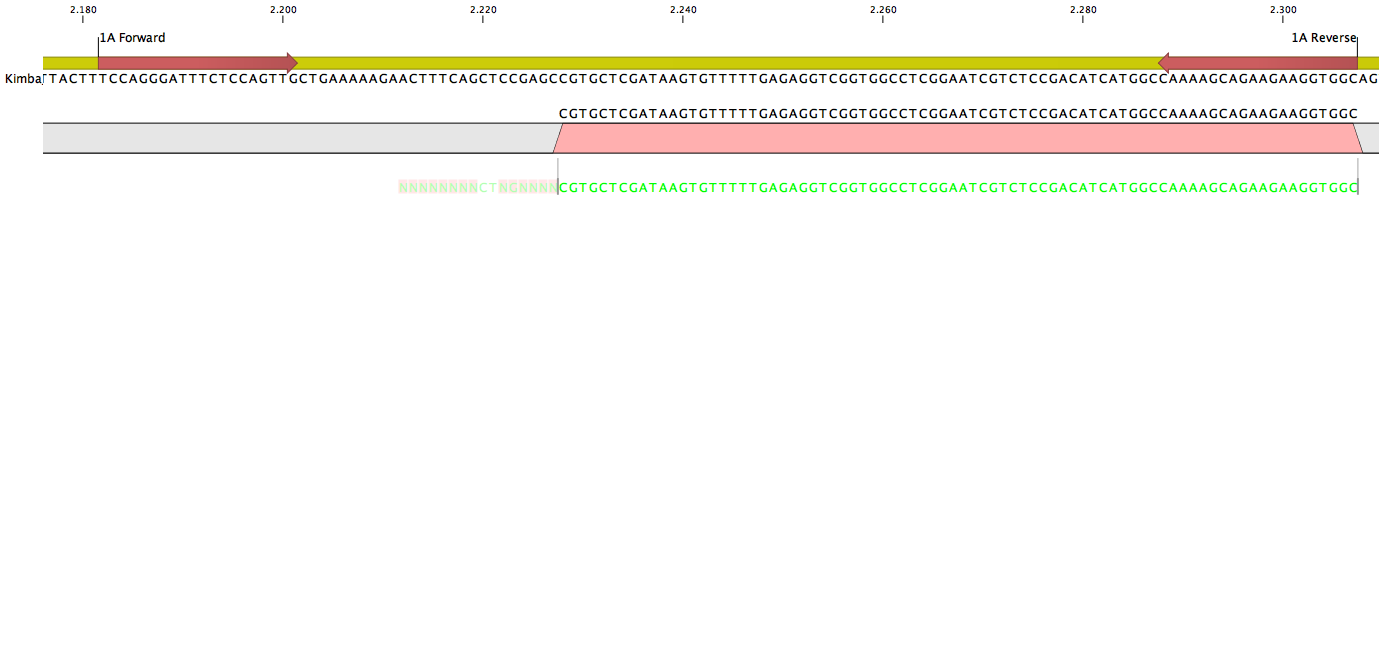

Supplement: S4 Fig — The PCR product was obtained by using 1A primers and DNA from EEHV1-positive, formaldehyde-fixed myocardium (S03-2084.6) as template. Cycle sequencing was done using the same primers. The obtained reverse sequence was aligned against the EEHV1A reference (Kimba). Mind: a forward sequence was not obtained. (TIF) [file pone.0176891.s004.tif]
